# Utility of micro-CT for dating post-cranial fractures of known post-traumatic ages through 3D measurements of the trabecular inner morphology

**DOI:** 10.1038/s41598-022-14530-1

**Published:** 2022-06-22

**Authors:** Alessia Viero, Lucie Biehler-Gomez, Carmelo Messina, Annalisa Cappella, Konstantinos Giannoukos, Guido Viel, Franco Tagliaro, Cristina Cattaneo

**Affiliations:** 1 Legal Medicine and Toxicology Unit, Department of Cardio-Thoraco-Vascular Sciences and Public Health, University of Padua, Padua, Italy; 2 Unit of Forensic Medicine, Department of Diagnostics and Public Health, University of Verona, P.le Scuro, 10, 37134 Verona, Italy; 3 Laboratorio Di Antropologia E Odontologia Forense (LABANOF), Sezione Di Medicina Legale, Department of Biomedical Sciences for Health, Università Degli Studi Di Milano, Via Mangiagalli 37, 20133 Milan, Italy; 4 IRCCS Istituto Ortopedico Galeazzi, Milan, Italy; 5 Department of Biomedical Sciences for Health, University of Milan, Milan, Italy; 6 Laboratorio Di Morfologia Umana Applicata, IRCCS Policlinico San Donato, San Donato Milanese, Italy; 7 Elettra-Sincrotrone Trieste S.C.P.A., SYRMEP Group, AREA Science Park, Strada Statale 14, 34149 Basovizza, Trieste, Italy; 8 World-Class Research Center “Digital Biodesign and Personalized Healthcare”, Sechenov First Moscow State Medical University, Moscow, Russia

**Keywords:** Musculoskeletal system, Trauma

## Abstract

Fracture dating is an issue at the forefront of forensic sciences. While dating fracture is crucial to understanding and verifying the chronology of events in cases of abuse and violent death, its application is the subject of considerable discussion in the scientific community, filled with limitations and difficulties. Current methods for fracture dating are mainly based on a qualitative assessment through macroscopy, microscopy, and imaging and subject to variations depending on the experience of the observer. In this paper, we investigated the potential of quantifiable micro-CT analysis for fracture dating. Five histomorphometric parameters commonly used for the study of the 3D bone trabecular microarchitecture with micro-CT were calculated based on nine fractures of known post-traumatic ages, including the degree of anisotropy, connectivity density, bone volume fraction, trabecular thickness, and trabecular separation. As a result, trends in the evolution of the microarchitecture of the bone relative to age of the callus could be identified, in particular concerning anisotropy, trabecular separation and connectivity density, consistent with the healing bone process. The findings obtained in this pilot study encourage further research in quantifiable parameters of the bone microarchitecture as they could represent useful features for the construction of objective models for fracture dating.

## Introduction

Trauma analysis in forensic casework is not only crucial because it can give important information for the personal identification of unknown corpses (especially in modern scenarios of mass migrations)^1–3^, but also because, through the accurate dating of bone fractures^4–6^, it can assist in testing witness statements and help understand the chronology of events prior to death^7,8^ in cases of violent death, abuse, maltreatment and torture^9^.

In forensic anthropological practice, the request to date skeletal fractures from autopsies or human remains is frequent, with particular reference first to the distinction between antemortem and perimortem fractures, and second to the estimation of the amount of time elapsed between the moment when the fracture occurred and the time of death. The accurate dating of bone fractures has become one of most important contributions of forensic anthropologists in legal medicine, including dry bones, where injuries may be preserved for a long time given the resistance of bone tissue to decomposition.

The estimation of the post-traumatic survival time (PTST) is based on the tissue healing response after traumatic tissue damage^10^ and requires knowledge not only of the biomechanics of bone fracture, but also of the bone remodeling system, patterns of growth and development, as well as the many variables involved in postmortem bone decay^11,12^.

However, despite the importance of the forensic assessment of the age of bone calluses, the available literature mainly consists of clinical studies^13–18^ performed by orthopaedists and radiologists more interested in the final stages of fracture healing as well as in the best approaches for improving fracture repair. By opposition, studies performed for forensic purposes, particularly looking at improving methods for ageing calluses, are scarce. Traditional anthropological methods are unable to solve the issue of the accurate dating of fractures, especially in the earliest stage of healing when the bone still possesses its elastic properties at the time of the fracture, but no mineralized signs of healing can be observed macroscopically. These difficulties also characterize the last stage of healing when the outer bone surface may not give a complete picture of the biology and inner structure of the remodeled fracture^19^.

A preliminary literature search conducted in the electronic databases Pubmed and Web of Science using a combination of free text protocols, such as “*fracture dating*”, “*fracture healing*”, “*fracture timing*”, “*bone callus*”, “*fracture repair*” and “*forensic*”, evidenced a limited number (n = 26) of forensic articles dealing with the study of fracture healing and dating in the dead. Most of them were based on skeletal remains, with only few works on post-mortem skeletal radiography on corpses compared to histopathologic analysis in pediatric forensic cases^20–23^, in which the detection and dating of infant fractures plays an important role in the diagnosis of the battered child syndrome^24,25^.

The application of non-destructive 3D imaging technologies to bone healing research, such as micro-computed tomography (micro-CT), has shown tremendous potential, particularly for fracture dating^26,27^. Yet, to date, only one qualitative and descriptive study has been reported to obtain a morphological bone description^28^, while quantitative assessments through micro-CT of the inner tridimensional trabecular microarchitecture of the bone aimed at dating post-cranial bone fractures have never been undertaken.

Therefore, the present experimental pilot study aims at evaluating the applicability of a high-resolution radiological technique such as micro-CT on dry bones to find useful parameters able to describe the trabecular microarchitecture for objective fracture dating.

## Material and methods

Given the high prevalence of rib fractures found during forensic autopsies, a sample of nine costal bone fractures was collected from six adult individuals autopsied at the Institute of Legal Medicine of Milan, in accordance with article 41 of the Italian National Police Mortuary Regulation (September 10, 1990; n° 285) and with authorization from the ASL (*Azienda Sanitaria Locale*—Local Health Authority). Among the individuals included in the study, five were males and one was female, with ages-at-death ranging between 39 and 55 years. No individual in the study presented diseases that might have interfered with fracture repair, such as osteoporosis or other metabolic bone disorders. Furthermore, all fractures were of traumatic origin, due to car accidents or aggressions, as inferable from medical records or witness testimonies, and of known post-traumatic ages: N1 (8 days), N5 (28 days), N4 (42 days), N3 (72 days), N2a, b, c, and d (86 days), and N6 (2 years).

During autopsies the flesh was mechanically removed from all bone samples which were then macerated in cold water until complete soft tissue removal.

Subsequently, a micro-CT analysis of the nine bone fractures was performed at *Elettra–Sincrotrone Trieste* using a custom-made high resolution radiological system based on a microfocus conventional source. The X-ray source, Hamamatsu L12161-07, was operating at 50 kV and current 400 μA at 20 W (focal spot size 20 μm) with 0.5 mm Al filter. The detector used was a Hamamatsu C11701DK-40, 2192 × 1776 pixels of isotropic pixel size of 120 μm. The object to source distance was 110 mm and the source to detector distance was 600 mm. The sample was rotating along its vertical axis at a constant speed of 0.225 ^o^/s and 1800 projections were acquired over 360^o^ in 30 min. The reconstruction was performed for an isotropic voxel size of 22 μm using NRecon version 1.7.0.4. by Bruker microCT 2012–2016. The scan was centered on the region of the bone callus, including as much as possible of the surrounding normal bone.

For each sample, four regions of interest were then selected by visual inspection: two in the callus region and two far from the fracture borders for adequate comparison of the measured parameters. Because in almost all cases no large homogenous region could be found for the definition of a Representative Elementary Volume (REV), it was decided to consider for the callus the maximum allowable volume (parallelepipedon) in the selected area and a similar amount for the normal bone. In general, the REV calculation of a heterogeneous porous structure like bone is far from resolved since many parameters (except porosity) need to be examined^29^. The study of a REV for a bone is related to its function^30^ and entire studies should be done if absolute parameters are sought^31^. In the present study, the choice of each REV was done only for comparative purposes amongst the trabecular regions of the bones. The chosen volumes were large enough, being a few orders of magnitude larger than the minimum trabecular spacing and thus they were assumed to be statistically representative of the bone history.

All areas containing useful and informative qualitative details about the callus age were selected by the forensic anthropologists and pathologists (AV, LBG and CC), who carefully hand-selected the regions under investigation, based on the analysis of the coronal and sagittal sections (22 µm thickness) obtained from the 3D dataset.

The process of reconstruction resulted in slices characterized by a histogram related to the intensities of the grey levels. The automatic segmentation of the cortical from the trabecular region was done based on the dual threshold technique for in vivo micro-CT bone analysis, namely Buie method^32^, as provided by Dragonfly software (academic license), Version 2020.1.1.809 [Windows] and where necessary the 3D viewer from ImageJ version 1.53c. The inputs to the method were the threshold for the enclosure of the entire bone region from the outer cortical surface to the trabecular and the threshold for the separation of cortical and trabecular skeleton. In our study the two thresholds were respectively 50 μm and 15 μm. For the first threshold we have used the Otsu method for separation of the bone from the non-bone areas. That threshold was 18,911 in grayscale values and was used to extract the periosteal surface. The second threshold was 9,617 in grayscale values that led to the successful segmentation of the trabecular areas from the cortical. Since the samples were all similar, those dual threshold values correspond to each one of our specimens. The selection of the thresholds took place after visual inspection of the segmentation quality from three independent users.

The given thresholds have the following explanation. The first one fills the inner area of the bone creating a mask including both cortical and trabecular regions. This threshold allows us to select both the cortical and trabecular region of interest. In case that the external cortical bone creates external cavity(ies) that do(es) not contain any trabecular region, this threshold allows for the omission of those areas. In other words, only volumes of the cortical bones that enclose a trabecular structure are used for the morphological analysis. The second threshold is important since it separates the cortical from the trabecular regions. Values greater than a specific value include part of the cortical area, while values less than this value lead to an underestimation of the trabecular region. For further reading on the overall process of the selection of masks for the overall and trabecular bone see previous research^32^.

The main features of the bones, including cortical porosity and trabeculae, were then preserved after applying the unsharp filter. The edges of each trabecula were greatly differentiated from the pores in order for the segmentation to be accurate and precise.

For the purpose of this study, the five histomorphometric parameters most commonly used for the study of the 3D bone trabecular microarchitecture with micro-CT^33^ were selected (Table 1), including the degree of anisotropy, connectivity density, BV/TV fraction [%] (bone volume fraction), trabecular thickness [mm] (TbTh) and trabecular separation [mm] (TbSp) of the segmented binary images. All these parameters were calculated using the BoneJ plugin in ImageJ^35^.

**Table 1:** Measurements of the histomorphometric parameters commonly used for the study of the 3D bone trabecular microarchitecture with micro-CT on nine fractures of known PTST.

	PTST	Degree of anisotropy		Connectivity density		BV/TV		Mean TbTh		Mean TbSp	
		HB	Callus	HB	Callus	HB	Callus	HB	Callus	HB	Callus
N1	8 days	0.77	0.79	191.69	120.69	0.10	0.10	6.74	6.36	48.09	55.87
N5	28 days	0.85	0.43	83.50	1488.38	0.12	0.42	8.03	7.41	51.63	12.64
N4	42 days	0.79	0.32	513.06	2519.81	0.12	0.34	7.19	7.22	40.77	17.13
N3	72 days	0.74	0.30	109.38	1011.88	0.15	0.38	8.74	10.02	43.15	21.55
N2A	86 days	0.80	0.39	39.31	1547.62	0.15	0.37	6.99	8.45	41.22	16.72
N2B	86 days	0.83	0.54	68.81	865.44	0.16	0.50	7.25	10.96	39.24	13.60
N2C	86 days	0.80	0.47	41.56	1366.12	0.14	0.47	8.37	9.52	43.99	15.63
N2D	86 days	0.81	0.42	26.69	1282.31	0.15	0.41	7.00	8.65	46.28	14.00
N6	2 years	0.80	0.73	109.63	169.94	0.13	0.30	6.36	11.08	29.83	29.42

*PTST* post-traumatic survival time, *HB* healthy bone, *BV/TV* bone volume fraction, *TbTh* trabecular thickness, *TbSp* trabecular separation; REVs 1 and 4 of healthy bone and REVs 2 and 3 of callus area were combined.

**Table:** 

Histomorphometric parameter	Description in BoneJ^34^
Degree of anisotropy	A measure of how highly oriented substructures are within a volume
Connectivity density	The number of connected structures in a network normalized by the volume of the sample
Bone volume fraction (BV/TV)	The volume of mineralized bone per unit volume of the sample
Trabecular thickness (TbTh)	A measure of the homogeneity of trabecular thickness
Trabecular separation (TbSp)	A measure of the homogeneity of trabecular separation

### Ethical approval

This article does not contain any studies with human living participants or animals performed by any of the authors. Informed consent for the rib samples was not needed (Police Mortuary Regulations, DPR 09/10/1990 n° 285, art. 41).

## Results

**Figure 1:**
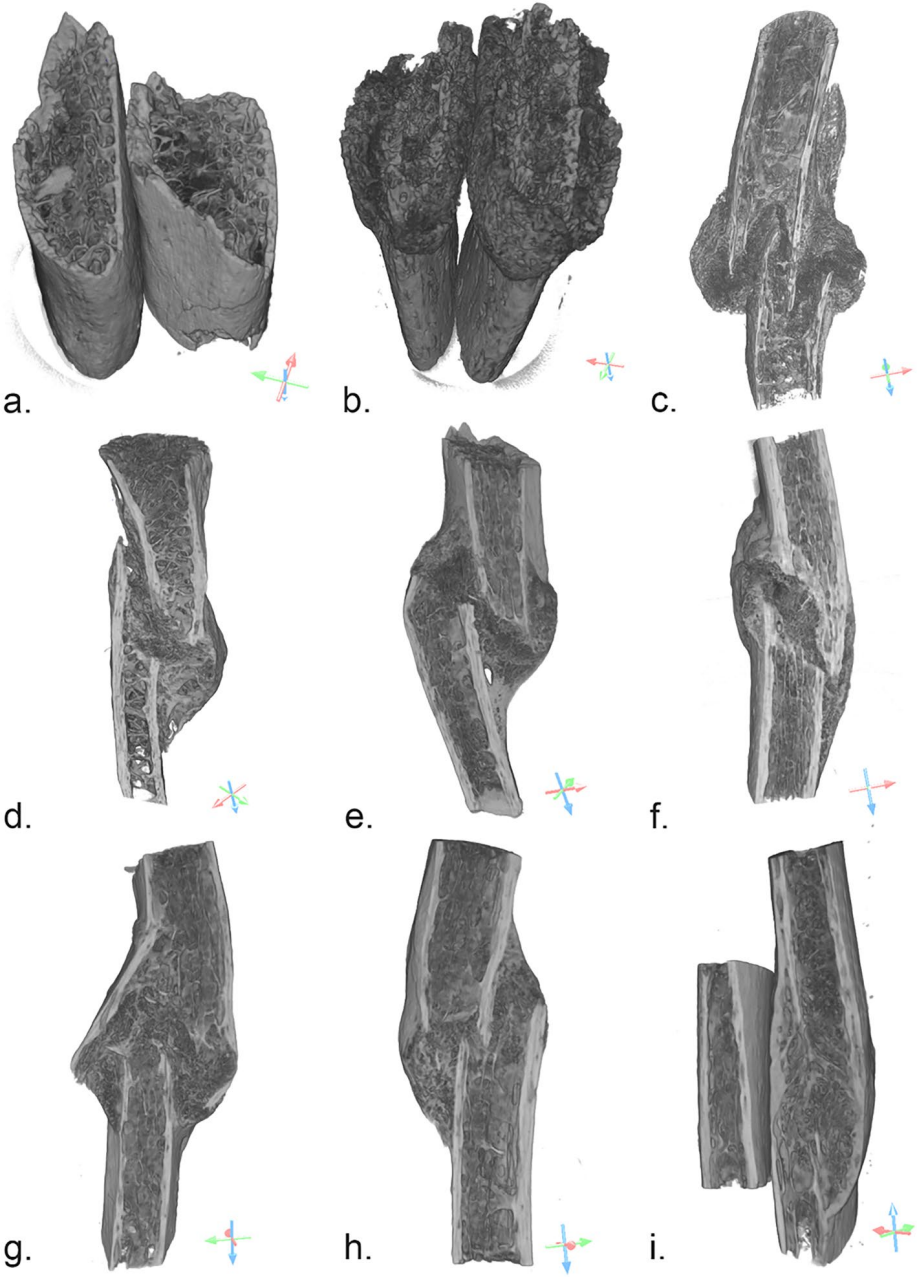
Selected areas of interest in the nine bone samples; (**a**): sample **N1**, 8 days old, areas of interest 1 and 3 with no signs of bone healing and sharp fracture margins next to the bony trabeculae, areas 2 and 4: healthy bone; (**b**): sample **N5**, 28 days old, areas of interest 2 and 3 with woven non-remodeled bone close to fracture line, areas 1 and 4: healthy bone; (**c**): sample **N4**, 42 days old, areas of interest 2 and 3 with exaggerated bulging callus accumulating at the periphery, areas 1 and 4: healthy bone; (**d**): sample **N3**, 72 days old, areas of interest 2 and 3 with exuberant woven bone formation, areas 1 and 4: healthy bone; (**e**): sample **N2A**, 86 days old, areas of interest 2 and 3 with denser woven bone, areas 1 and 4: healthy bone; (**f**): sample **N2B**, 86 days old, areas of interest 2 and 3 with signs of advanced healing, including lamellar oriented trabeculae and a smoother appearance of the cortical regions, areas 1 and 4: healthy bone; (**g**): sample **N2C**, 86 days old, areas of interest 2 and 3 with lamellar and woven bone, areas 1 and 4: healthy bone; (**h**): sample **N2D**, 86 days old, areas of interest 2 and 3 with dense woven callus and complete bridging on the left side, areas 1 and 4: healthy bone; (**i**): sample **N6**, 2 years old, areas of interest with 2 and 3 remodeled lamellar bone, areas 1 and 4: healthy bone. Dragonfly software, Version 2020.1.1.809 (academic licence) from Object Research Systems (ORS) Inc, Montreal, Canada, 2020 (software available at http://www.theobjects.com/dragonfly) was used to generate the volume renderings.

The 3D measurements of the trabecular inner morphology of the bone samples analyzed were performed on the four areas of interest selected (Fig. 1): two located in the callus and two in areas more distant to it (referred to as healthy bone—HB). Healthy bone measurements were performed to obtain “normal” values to contextualize callus measurements. The bones of Fig. 1 consist of orthographic projections of the reconstructed volumes and the floating scale bars denote the relative sizes of each specimen. Areas of HB that also included cortical bone were used to calculate the 3D bone trabecular microarchitecture only.

The data obtained, which correspond to the regions of the trabecular bone (REVs) from Fig. 1, are reported in Table 1 to evaluate changes in the trabecular microarchitecture with the PTST both at the site of the callus and in areas of healthy bone.

**Figure 2:**
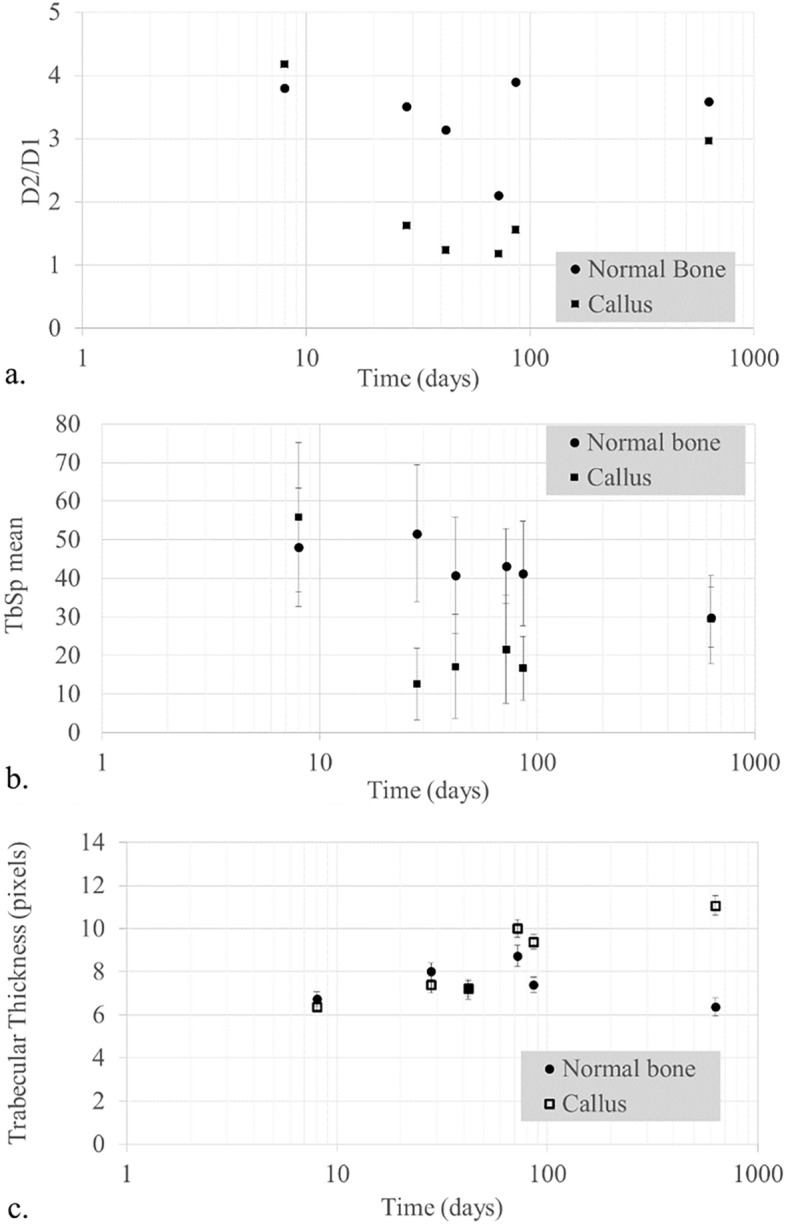
Degree of anisotropy (**a**), trabecular separation (**b**) and trabecular thickness (**c**), (**a**): ratio between the medium (D2) and the shorter ellipsoid axis (D1) in order to test the degree of anisotropy in the transverse plane of the rib both in the callus and in areas distant from it ; (**b**): ratio of the mean value of the trabecular separation (TbSp) and its standard deviation both in the callus and in areas distant from it; (**c**): time-evolution of trabecular thickness of the callus and the normal bone away from the fracture. Values for N2 were averaged. Graph realized with Microsoft Excel.

The degree of *anisotropy* is used to quantify the directionality of the stresses of trabecular bone and informs on whether the trabeculae have a certain stress–strain orientation. Figure 2a presents the time-evolution of anisotropy in terms of the ratio between the diameters used to fit an ellipsoid within a pore of the trabeculae. That ratio is represented as D2/D1 and is in fact analogous to the anisotropy.

The lower the ratio D2/D1 is, the more isotropic the trabeculae become and thus, the less oriented they present. The initial assumption that the trabecular bone was mainly oriented along the rib longitudinal axis was demonstrated by the higher D2/D1 ratios in the areas of the bone far from the callus. The use of the ratio between the longer (D2) and the shorter ellipsoid axis (D1) was used to evaluate and test the degree of anisotropy in the transverse plane of the rib itself and the observed trend appeared even more clearly, as represented in Fig. 2a.

The use of this particular ratio better reflects the directionality of the anisotropy given that the value of the degree of anisotropy (DA) does not reflect a particular orientation. D1 and D2, as the eigenvalues of each examined volume, represent the anisotropy not only as a value but also in the direction of the increasing anisotropy. That direction was oriented from the healthy bone towards the fractured bone. The current study demonstrated the first step towards more detailed research in the calculation of anisotropy in fractures for forensic sciences.

The inner microarchitecture of the callus appeared to remain as anisotropic as the healthy bone in the early stages of fracture healing (N1). Then the trabeculae were found to become increasingly isotropic during the intermediate stages of healing (N5-4–3), before becoming anisotropic in the last stages (N2-6). An important observation is that the trabeculae after 2 years appeared to obtain less anisotropic states than the initial microstructure.

*Trabecular separation (TbSp)*, represented in the graph as the mean value along with standard deviation (Fig. 2b), appeared to follow a similar behavior as the degree of anisotropy. Figure 2b shows that the error bars overlap for various times. This can be explained from parts of the callus that created structures similar to the normal bone. For the latest specimen, it appeared that the TpSp of the callus and normal bone coincided, but with TbSp less than the initial specimens.

Considering that the first point (fracture N1) refers to a sample that does not show any visible callus, we see low values at the beginning of the healing process (N5) that are increasing with the PTST (N4-3–2-6). The *TbSp* of the callus after two years of healing appeared to show a denser microstructure than the initial specimen (N1). However, the trabeculae are characterized by higher *TbSp* values than the initial structure (N1). It is notable that the healing process up to two years did not allow the callus to obtain similar *TbSp* as the bone away from the fracture. These results show that the callus starts growing with a lateral isotropic trabecular fine mesh and becomes more oriented with larger spacing during the healing process, but never reaches the initial microstructure.

**Figure 3:**
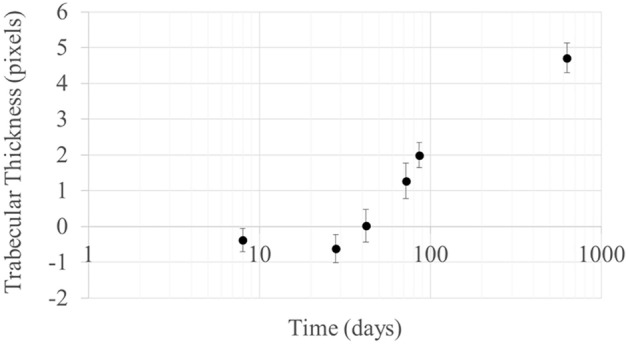
Normalized trabecular thickness Tb.Th(callus)-Tb.Th(healthy) per sample. Graph realized with Microsoft Excel.

Measurements of the *trabecular thickness*, which represents the average thickness of the trabeculae relative to the age of the callus is shown in Fig. 2c. The increasing thickness of the trabeculae at the callus after two years is evident, in contrast to the constant thickness at the trabeculae far from the fracture site. Figure 3 shows the normalized trabecular thickness as expressed by the values of the subtraction Tb.Th(callus)-Tb.Th(healthy) in time. Negative values seem to correspond to early times of the fracture while positive values appear to represent fractures with increased degree of healing.

**Figure 4:**
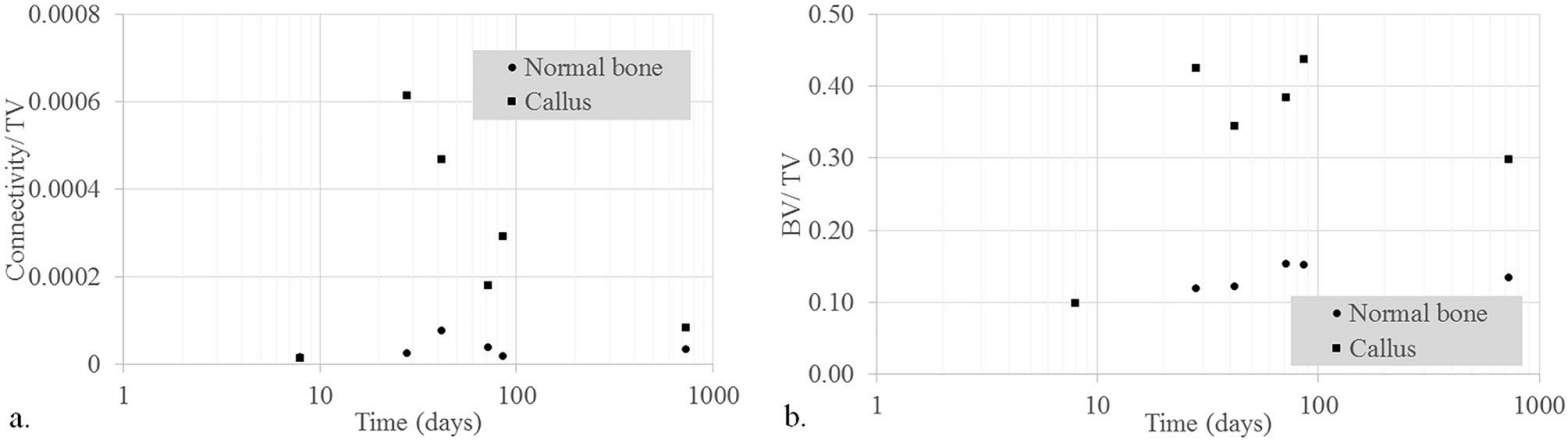
Connectivity density (**a**) and bone volume fraction (**b**), (**a**): ratio of the connectivity and total volume both in the callus and in areas distant from it; (**b**): bone volume fraction in the callus and in areas distant from it. Values for N2 were averaged.

Another parameter which was examined is *connectivity density*, which was used to estimate the number of connected nodes in the trabecular network. This parameter is represented in Fig. 4a as the ratio of the connectivity and total volume. The intermediate stages of the healing process showed a six-fold to two-fold increasing of the degree of connectivity density (N5-4-3-2), while after two years (N6) the trabeculae appeared with similar connectivity density as the initial bone. In our study, we were not able to detect whether old or new nodes had been restored or created, respectively. The healing process seemed to have proceeded with a highly interconnected trabecular microstructure of the callus, which is in line with the small mean of trabecular spacing.

Finally, *bone volume fraction (BV/TV)* presented in Fig. 4b shows that the time evolution of healing of the callus creates a higher bone density in the fracture, while the volume of the trabeculae away from the fracture remains constant.

**Figure 5:**
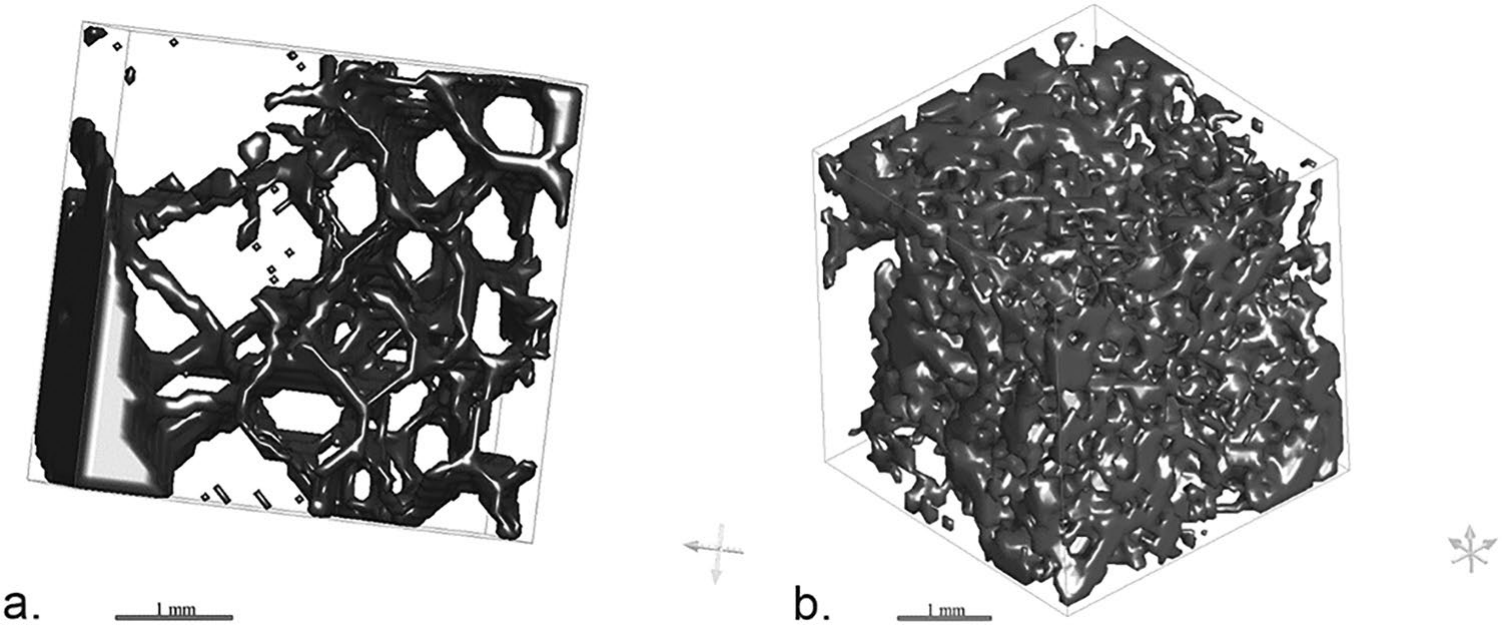
Contour meshes (cubic boxes with size 4 mm) of the trabecular microarchitecture of the bone far from the callus (healthy bone—**a**) and of a callus with a PTST of 86 days (**b**). Dragonfly software, Version 2020.1.1.809 (academic licence) from Object Research Systems (ORS) Inc, Montreal, Canada, 2020 (software available at http://www.theobjects.com/dragonfly) was used to generate the volume renderings.

The different aspect of the trabecular microarchitecture of the bone far from the fracture (referred to as healthy bone) and within the callus can also be appreciated through 3D rendering, as illustrated in Fig. 5. As a result, the 3D rendering clearly shows how the distance between the trabeculae is greater in the inner structure of the bone far from the fracture than within the callus at an intermediate stage of healing (86 days). Graph realized with Microsoft Excel.

## Discussion

This study aimed to test the potential of 3D histomorphometric parameters for the accurate determination of fracture dating. This pilot study steered toward bridging the gap in the current literature regarding PTST estimation for forensic caseworks.

Among the studies based on skeletal remains, Barbian et al.^36^ and Maat et al.^37^ reported that a minimal period of several days is required for the gross detection of initial osseous evidence of a healing response^12^. The inability to solve the issue of the accurate dating of fractures with traditional anthropological methods has driven forensic researchers over the last decades to test the potential of both histology^8,24,25^ and new radiological tools^38^ to identify vital processes in dry bones, even when macroscopical changes are not yet visible^2,37,39^.

Histology is considered the most accurate method for dating fractures and the most precise in the early healing stages, when the gross examination of the outer bone surface does not give a complete picture of the biology of the tissue’s reaction^2,8,19,37,39,40^. Histology proved to be superior to both conventional radiology (CR) and CT scanning^10^ but it is also a destructive method that requires specific training for interpretation^17,25^.

Consequently, new high-resolution imaging and minimally destructive methods have been studied for fracture dating^2,37,39,41,42^. Cappella et al.^38^ explored the potential of Cone-Beam CT (CBCT) for fracture analysis, showing that this imaging technique provided unique information on the different degrees of mineralization and a better visualization of the inner and outer callus structure and fracture line. Steyn et al.^26^ and Delabarde et al.^27^ investigated the potential of micro-CT for cranial fracture dating, another sensitive and high-resolution imaging technique widely used for clinical purposes in animal models^15,43–51^, and argued that the technique may be a powerful complement to histological analysis, but no quantitative analysis was performed. Finally, Viero et al.^28^ published a descriptive study showing the potential of qualitative evaluation of micro-CT sections for fracture dating, a study that was conducted on the same post-cranial bone fractures examined in the current work.

In light of the promising results of the three aforementioned forensic studies^26,27^, this pilot study represents a microstructural investigation using micro-CT imaging technology for the evaluation of post-cranial fractures from a quantitative point of view. Five histomorphometric parameters were selected to describe changes in the trabecular microarchitecture relative to the PTST.

As a result, micro-CT highlighted differences in the microarchitectural parameters between the callus and the non-fractured bone, mainly related to the degree of anisotropy and the spacing between the trabeculae.

The degree of anisotropy^52^ is a parameter that is used to numerically quantify the trabecular directions in a specific bone segment, pointing to whether there is a specific orientation of the trabeculae or if they are randomly arranged. In the samples analyzed, a clear difference emerged in the degree of anisotropy between the callus and the healthy bone in the intermediate stage of healing (N5-4-3). In fact, the newly formed callus presented a progressively more isotropic microstructure (i.e., a more anarchic architecture). This finding is in line with the current literature related to the formation of woven bone, which is characterized by collagen fibers (early stage) and bony trabeculae (later stages) irregularly arranged, showing no specific orientation related to cortical bone and vascular spaces^53^. With callus maturation, this anarchic organization is replaced over time by organized lamellar tissue.

Similarly, low values of trabecular separation (TbSp–N5) and an increase of connectivity density in the intermediate stages of callus formation (N5-4-3-2) was observed. This is equivalent to a callus showing less space within the newly formed trabeculae and collagen fibers as well as a higher connection between trabeculae in callus formation. Again, this is in line with the healing process of bone. In fact, the beginning of callus formation is characterized by a less dense woven bone mixed with cartilaginous and granulation tissue, which is progressively resorbed in favor of denser woven and lamellar bone^17^.

It is important to note that at early stages of fracture repair, mineralization may not yet be detectable by micro-CT. Therefore, results in the callus area at this stage (N1, PTST of 8 days) are probably from the trabecular bone itself and not specifically from the callus; this may explain why the results are so similar to those of the “healthy bone”.

Nonetheless, apart from the peculiarities of the earliest stages of fracture repair, our preliminary results obtained through quantitative micro-CT measurements of the 3D trabecular microstructure of bone calluses of known ages suggest the existence of trends for some parameters of trabecular microstructure relative to the age of the callus. These parameters included the degree of anisotropy, connectivity density and trabecular spacing, and could represent useful features for the construction of objective and quantitative models for fracture dating.

Given the variability of the inner structure of bone calluses, which can simultaneously present different healing features in different parts of the same callus, and the lack of previous quantitative studies on this topic, the main challenges when dealing with this kind of analysis consist are twofold: (1) the selection of the most representative trabecular bone volume of interest (REV) within the calluses and as “healthy bone” and (2) the identification of the most suitable histomorphometric parameters to be examined. Furthermore, the variability between calluses may be related to specific factors, which need to be considered. On the one hand, variability is related to the smaller semi-homogeneous volumes of the callus (compared with the trabecular spacing), due to the different types of fractures. In fact, certain fractures may be found with impacted cortical fragments within the callus, frayed opposed margins, or they can eventually evolve with restored cortical margins. On the other hand, variability may be related to the intrinsic biomechanics of the traumatic and healing process, as both compression and tension regions may coexist in the same callus.

Further points of research emanating from our study include the consideration of the maximum volume, which implied the loss of the fine structure, but this was a conscious choice for this pilot study which aimed to find synthetic parameters to describe a complex structure. However, the comparison between the inner characteristics of bone far from the fracture and of the callus is self-consistent since the areas analyzed came from the same bone samples.

In relation to the present study, critical consideration could also be expressed about the small sample size, as well as about the lack of choice of statistically homogeneous groups of samples around the fracture. Together with intra- and interpersonal variability, these are factors that do not permit any definitive statements about the dating of a bone, but only suggestions based on the microstructure of the trabeculae. In other words, our methodology can be used as a guiding tool for the micro-CT study of any type of fracture based on simple microstructural measurements of the trabeculae. Overall, the present findings suggest that healing enhances trabeculae mineralization (TbTh increases), creating more trabeculae nodes (connectivity increases) that first expanded isotropically (D2/D1 decreases) and then created a more compact microstructure over time as seen from the positive differences of the Tb.Th(callus)-Tb.Th(healthy bone). It would be interesting to extend the investigation to other types of bones to confront and strengthen our results.

Finally, despite the strengths and potential of the technique for fracture dating, it is important to note that micro-CT can only analyze small calluses, no longer than 2–3 cm and no wider than 1.5 cm. Thus, to be informative, this technique should only be applied to bone calluses which can be fully scanned. Conversely, large calluses should be sectioned but the heterogeneity of the internal structure of the callus itself could cause an important bias for the analysis of the sections.

## Conclusion

In case of skeletal remains, the need to apply new high-resolution imaging methods for a more accurate and objective analysis of the degree of fracture healing led to test micro-CT for the longitudinal evaluation of post-cranial bone fractures of known PTSTs not only because of its high resolution, but also in light of the promising results of three previous forensic studies on this topic.

The present research can be considered a preliminary study, but surely demonstrates the potential of micro-CT for the construction of objective fracture dating models using quantitative measurements of relevant histomorphometric parameters, therefore laying the groundwork for further studies on this topic.

Conversely, the sample size should be expanded to carry out an adequate statistical analysis and the sample itself should be homogeneously grouped. Nevertheless, these are some of the most common intrinsic limitations in the forensic research on this topic, since, because of interpersonal variability and the obvious limitations of the available cases, it is difficult to collect large numbers of calluses and to select homogeneous groups of cadavers.

## Data Availability

The volume renderings and data calculations for this paper were generated using Dragonfly software (academic license), Version 2020.1.1.809 [Windows]. Object Research Systems (ORS) Inc, Montreal, Canada, 2020; software available at http://www.theobjects.com/dragonfly.
